# Ubiquinol decreases hemorrhagic shock/resuscitation‐induced microvascular inflammation in rat mesenteric microcirculation

**DOI:** 10.14814/phy2.12199

**Published:** 2014-11-20

**Authors:** Qiuhua Shen, Naomi Holloway, Amanda Thimmesch, John G. Wood, Richard L. Clancy, Janet D. Pierce

**Affiliations:** 1 School of Nursing, University of Kansas, Kansas City, Kansas, USA (Q.S., A.T., J.D.P.); 2 Department of Surgery, University of Kansas, Kansas City, Kansas, USA (N.H., J.G.W.); 3 Department of Molecular and Integrative Physiology, University of Kansas, Kansas City, Kansas, USA (J.G.W., R.L.C., J.D.P.)

**Keywords:** Leukocyte adherence, mast cell degranulation, reactive oxygen species, ubiquinol, vascular permeability

## Abstract

Hemorrhagic shock (HS) is a leading cause of death in traumatic injury. Ischemia and hypoxia in HS and fluid resuscitation (FR) creates a condition that facilitates excessive generation of reactive oxygen species (ROS). This is a major factor causing increased leukocyte‐endothelial cell adhesive interactions and inflammation in the microcirculation resulting in reperfusion tissue injury. The aim of this study was to determine if ubiquinol (coenzyme Q10) decreases microvascular inflammation following HS and FR. Intravital microscopy was used to measure leukocyte‐endothelial cell adhesive interactions in the rat mesentery following 1‐h of HS and 2‐h post FR with or without ubiquinol. Hemorrhagic shock was induced by removing ~ 40% of anesthetized Sprague Dawley rats' blood volume to maintain a mean arterial blood pressure <50 mmHg for 1 h. Ubiquinol (1 mg/100 g body weight) was infused intravascularly in the ubiquinol group immediately after 1‐h HS. The FR protocol included replacement of the shed blood and Lactate Ringer's in both the control and ubiquinol groups. We found that leukocyte adherence (2.3 ± 2.0), mast cell degranulation (1.02 ± 0.01), and ROS levels (159 ± 35%) in the ubiquinol group were significantly reduced compared to the control group (10.8 ± 2.3, 1.36 ± 0.03, and 343 ± 47%, respectively). In addition, vascular permeability in the control group (0.54 ± 0.11) was significantly greater than the ubiquinol group (0.34 ± 0.04). In conclusion, ubiquinol attenuates HS and FR‐induced microvascular inflammation. These results suggest that ubiquinol provides protection to mesenteric microcirculation through its antioxidant properties.

## Abstract (precis)

Fluid resuscitation following hemorrhagic shock can cause reperfusion injury. Administration of ubiquinol prior to fluid resuscitation reduced microvascular inflammation in the rat mesentery microcirculation by decreasing leukocyte adherence, mast cell degranulation, vascular permeability, and reactive oxygen species levels.>

## Introduction

Hemorrhagic shock (HS) leads to decreased tissue perfusion, resulting in the inadequate delivery of oxygen and nutrients that are required for cellular function. The ischemia and hypoxia in HS and fluid resuscitation (FR) creates a condition that facilitates excessive production of reactive oxygen species (ROS). This is a major factor causing increased leukocyte‐ endothelial cell adhesive interactions and inflammation in the microcirculation resulting in reperfusion tissue injury. Previous studies have shown that systemic hypoxia can lead to rapid microvascular inflammatory responses, including increased number of adherent leukocytes within mesenteric venules (Wood et al. ^24^). Increased ROS levels and leukocyte adherence were observed in systemic hypoxia caused by HS (Childs et al. ^10^). Restoration of oxygen delivery after a certain period of ischemia can lead to reperfusion injury by potentiating the generation of ROS, which further intensifies leukocyte‐endothelial cell adhesive interactions resulting in increased number of adherent leukocytes, mast cell degranulation, and vascular permeability to albumin (Cerqueira et al. ^8^).

As an endogenously synthesized and lipid‐soluble antioxidant, ubiquinol is the active form of coenzyme Q10. It is found in almost every cell in the body and plays two key functions: (1) electron transport in the mitochondrial respiratory chain, and (2) scavenging ROS. Compared to ubiquinone – its oxidized form, ubiquinol has two electrons that can neutralize the unpaired electron of ROS. It protects cell membranes from lipid peroxidation and promotes regeneration of other antioxidants such as Vitamin C and Vitamin E. Previous studies have shown that administering antioxidants such as superoxide dismutase, catalase, or lipoic acid in systemic hypoxia can scavenge ROS and subsequently reduce microvascular inflammatory responses (Wood et al. ^23^). However, to our knowledge, there are only three studies investigating the use of ubiquinone (the oxidized form of coenzyme Q10) in HS (Aoyagi ^2^; Hatano ^15^; Yamada ^25^). None of the three studies examined the effects of ubiquinone on microvascular inflammation when administered before FR following HS.

The purpose of the study was to determine if ubiquinol decreases microvascular inflammatory responses following HS and FR, using an HS rat model. We examined if intravascularly administered ubiquinol following 1‐h HS would: (1) decrease ROS levels in microvascular circulation; (2) reduce leukocyte‐endothelial cell adhesive interactions; (3) decrease mast cell degranulation; and (4) attenuate the increase in vascular permeability during the 2‐h period after FR.

## Materials and Methods

The experimental and surgical protocol involving animals was submitted to and approved by the Animal Care and Use Committee at the University of Kansas Medical Center (KUMC). The KUMC animal care facility is fully accredited by the American Association for the Accreditation of Laboratory Animal Care. All the surgical and experimental procedures followed the guidelines established by the National Institutes of Health and the Public Health Services Policy on the humane use and care of laboratory animals. Animals were allowed to acclimate at least 48 h prior to the experiments. They were housed in pairs and maintained in twelve hours light‐dark cycles and provided standard rat chow and water ad libitum.

### Surgical procedures

Male adult Sprague‐Dawley rats (Harlan, Indianapolis, IN) were used in the study. The rats were 10–14 weeks old with a mean weight of 378 g. Rats were anesthetized by intramuscular injection of sodium pentobarbital (50 mg/kg) in both rear legs. Periodic nail pinches were performed to ensure the adequacy of anesthesia. Supplemental sodium pentobarbital (25 mg/kg) was administered as needed during the experiment to maintain a surgical plane of anesthesia. After the surgical plane was achieved, a tracheostomy was performed using polyethylene tubing (PE‐240) to facilitate spontaneous breathing of room air. The right carotid artery was cannulated with a polyethylene catheter (PE‐50) and connected to a Blood Pressure Analyzer 400 (Micro‐Med, Louisville, KY) to measure arterial blood pressures. The left femoral artery was also cannulated with a PE‐50 catheter and used for inducing HS and administering FR and solutions.

The rat was placed on a homeothermic blanket system connected to an intrarectal temperature probe to maintain body temperature approximately 37°C throughout the experiment. A 2‐cm midline abdominal incision was made using an electrocautery (Harvard Apparatus, Holliston, MA). The animal was then placed on a Plexiglas sheet laying laterally on its right side. A section of small intestine was carefully exteriorized and positioned over a glass coverslip on the Plexiglas sheet to view mesenteric venules. A small piece of Saran wrap was placed on top of the mesentery to prevent drying of the tissue.

A mesenteric venule was selected and visualized using an Axiovert inverted microscope (Carl Zeiss Microscopy GmbH, Jena, Germany). The venule selected met the following criteria: (1) straight, unbranched at least 100 μm in length; (2) diameter between 20 and 40 μm; (3) no adjacent venules within 100 μm; and (4) less than two adherent leukocytes within the 100 μm segment of the venule. A video caliper (Microcirculation Research Institute, College Station, TX) was used to measure venular diameter.

### Experimental protocols

There was a 30‐min post‐surgery control period prior to the experiment. Hemorrhagic shock was induced by removing ~ 40% of blood volume to maintain a mean arterial blood pressure (MABP) <50 mmHg for 1 h. The shed blood was collected in a heparin‐coated syringe and kept at room temperature until reinfusion. Rats were randomly assigned to either the control or ubiquinol group. The rats in the ubiquinol group were administered via the femoral catheter the QH Liposomal Ubiquinol solution (1 mg/mL, 1 mg/100 g of body weight) (Tishcon Corporation, Westbury, NY) prior to the FR with shed blood and Lactated Ringer's (twice the volume of blood removed). Twice the amount of LR was used since it has been a recommendation used in clinical settings for FR following HS (American College of Surgeons ^1^; Cannon ^6^). The animals in the control group were resuscitated with shed blood and Lactated Ringer's only. Fluid resuscitation was completed within 5 min. Blood pressures (MABP and systolic/diastolic blood pressures) were continuously monitored and recorded every 10 min throughout the experiments for 2 h following FR. Arterial blood values were measured at baseline, HS, and at the end of the experiment using I‐STAT (Abbott Laboratory, Abbott Park, IL).

### Measurement of leukocyte adherence

Leukocyte adherence was assessed and recorded for 1 min every 10 min throughout the experiment with a digital Sony video recorder (Panasonic, Osaka, Japan) connected to the inverted microscope. The total number of adherent leukocytes was determined by counting the number of leukocytes that remained stationary for longer than 30 sec in each minute within the 100‐μm venule during playback of the videotape (Wood et al. ^24^). Since leukocyte adherence can be affected by the venule wall shear rate, it was measured by the following method. The center‐line red blood cell (RBC) velocity in the venule was assessed by an optical Doppler velocimeter (Microcirculation Research Institute, College Station, TX). Venular wall shear rate was calculated as (8 × center‐line RBC velocity)/(1.6 × venular diameter).

### Measurement of mast cell degranulation

Ruthenium red (0.001%) was superfused on the mesentery every 15 min to evaluate the degree of mast cell degranulation. This dye permeates only degranulated mast cells resulting in a pink color. Images of 10 intact mast cells in the mesenteric microcirculation around the selected venule were recorded during the 30‐min control period. Images of the same 10 mast cells were recorded at the end of the experiment. Mast cell images were converted to digitized grayscales and then phase inverted. The relative light intensity of each mast cell within the field of view was measured. The mast cell degranulation index was calculated as the ratio of light intensities at the end of experiment to the control period. The higher mast cell degranulation index indicates a higher degree of mast cell degranulation.

### Measurement of vascular permeability

Vascular permeability of the mesenteric venule was estimated using fluorescein isothiocyanate (FITC)‐labeled bovine albumin. Increased permeability to the FITC‐albumin indicated increased vascular permeability. The FITC‐labeled bovine albumin (50 mg/kg) was injected via the femoral artery catheter to anesthetized rats 30 min before each measurement of vascular permeability at baseline, HS, 1‐h and 2‐h FR. An intensified charge‐coupled device (ICCD) camera (Hamamatsu Photonics, Shizouka, Japan) was used to record fluorescence intensity of FITC‐albumin at an excitation wavelength of 420–490 nm and an emission wavelength of 520 nm. To avoid photobleaching, duration of fluorescence recording was less than 15 sec in a given area. The fluorescence intensity was obtained from three contiguous areas within the venule and from three areas in the adjacent perivenular regions at baseline, following HS, 1‐h and 2‐h FR using NIH Image 1.62 (National Institute of Health, Bethesda, MD). Each individual area was a circle with the same diameter as that of the venule (Casillan et al. ^7^). The averages of the fluorescence intensities within and outside the venule were calculated as intravascular or extravascular fluorescence intensities, respectively. The vascular permeability index was calculated as the ratio of extravascular to intravascular fluorescence intensities. A higher index indicates higher vascular permeability.

### Measurement of microvascular ROS levels

Dihydrorhodamine (DHR), an oxidant‐sensitive probe, was used to measure ROS levels within the venular wall of mesentery using the ICCD camera. The mesentery was superfused with DHR (200 μL) every 10 min throughout the experiment. The DHR fluorescence intensities along the venular wall were recorded every 30 min with the duration of recordings being less than 15 sec. The intensity of the fluorescent signal was measured by NIH Image 1.62 in five adjacent circles of 5 μm diameter along the venule. The same field of view was monitored throughout the experiment. The average DHR fluorescence intensity was collected at baseline, HS, and every 30 min post FR. Values for DHR fluorescent intensity at HS, 30‐min, 60‐min, 90‐min and 120‐min post FR were expressed relative to baseline intensity, which was defined as 100%.

### Statistical analyses

All values were expressed as means ± standard error of the mean (SEM). Independent t‐test was used to compare the differences in blood pressures, leukocyte adherence, mast cell degranulation, vascular permeability, and ROS levels between the ubiquinol and control groups. Paired *t*‐tests were performed to compare within group differences at various time periods throughout the experiments in blood pressures, leukocyte adherence, mast cell degranulation, vascular permeability and ROS levels with Bonferroni adjustment for multiple comparisons. All data analyses were performed in SPSS (version 20.0, IBM Corporation, Armonk, NY). Significance level was set as *P*‐value <0.05.

## Results

Frequent measurements of arterial blood gases were similar to our previous HS experiments with and without ubiquinol (Bennetts et al. ^4^). In summary, arterial blood pH was unchanged in both groups throughout the experiment, as a result of hyperventilation compensating for the metabolic acidosis.

### Hemodynamics

There were no significant differences in blood volume removed to induce HS between the control (9.1 ± 0.6 mL) and ubiquinol groups (10.3 ± 0.7 mL) (*P *>**0.05). Table 1 summarizes the blood pressure data at baseline, HS, and 2‐h post FR for the control and ubiquinol groups. There were no significant differences in blood pressures at baseline, HS, and 2‐h post FR between the control and ubiquinol groups. Compared to baseline, there were significant decreases in all arterial blood pressure measurements within both groups at shock, and 2‐h post FR (*P *<**0.05).

**Table 1.** Blood pressures in the control and ubiquinol groups at baseline, hemorrhagic shock, and 2 h after fluid resuscitation.

	Control	Ubiquinol
Baseline		
SBP (mmHg)	158 ± 2	154 ± 3
DBP (mmHg)	108 ± 7	104 ± 3
MAP (mmHg)	134 ± 4	130 ± 2
Hemorrhagic shock		
SBP (mmHg)	71 ± 3^1^	75 ± 2^1^
DBP (mmHg)	34 ± 2^1^	32 ± 1^1^
MAP (mmHg)	46 ± 1^1^	46 ± 1^1^
2 h after fluid resuscitation		
SBP (mmHg)	138 ± 4^1^^,^^2^	139 ± 5^1^^,^^2^
DBP (mmHg)	90 ± 3^1^^,^^2^	92 ± 5^1^^,^^2^
MAP (mmHg)	113 ± 3^1^^,^^2^	114 ± 5^1^^,^^2^

SBP, systolic blood pressure; DBP, diastolic blood pressure; MAP, mean arterial blood pressure.

Data are presented as mean ± SEM; *n* = 10.

^1^ Significantly different from baseline (*P* < 0.05).

^2^ Significantly different from shock (*P* < 0.05).

### Leukocyte adherence

**Figure 1.**
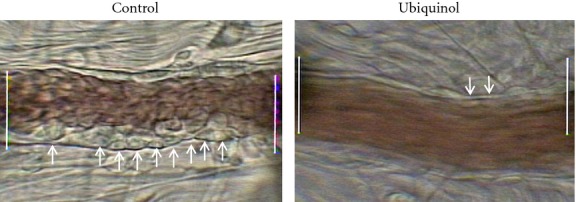
Exemplar images of mesenteric venules showing leukocyte adherence at 2 h after fluid resuscitation in the microcirculation of rats in the control and ubiquinol group following hemorrhagic shock. The arrows indicate adhered leukocytes. The distance between the white lines is 100 μm.

**Figure 2.**
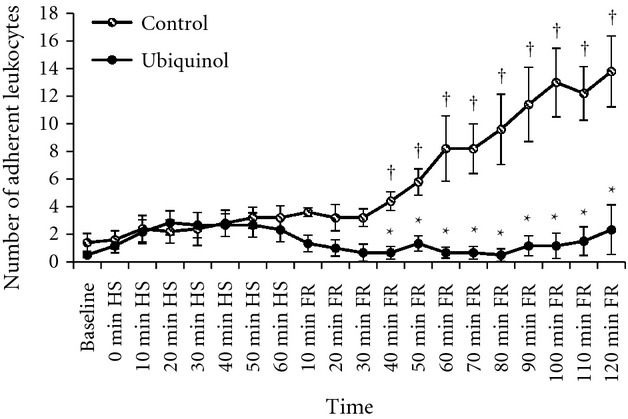
Number of adherent leukocytes per 100 μm of mesenteric venule in the control (*n* = 6) and ubiquinol (*n* = 6) groups at baseline, during hemorrhagic shock (HS), and fluid resuscitation (FR). ^†^Significantly different from baseline (*P* < 0.05). *Significantly different from the control group (*P *<**0.01).

Figure 1 illustrates exemplar images of leukocyte adherence in the mesenteric venules 2‐h post FR in a control rat and an ubiquinol rat. Less than two adherent leukocytes were observed during the 30‐min control period in both groups. The time course of leukocyte adherence is presented in Figure 2. A modest but insignificant increase in the number of adherent leukocytes during HS occurred in both groups. Beginning at 40 min of FR, the number of adherent leukocytes in the control group rapidly increased, reaching almost 10 times the baseline value at the end of the experiment. In contrast, leukocyte adherence in the ubiquinol group remained at baseline level throughout the FR period. The average venular diameter in the ubiquinol group was 27 ± 2 μm, which was not significantly different from that in the control group (23 ± 1 μm). In addition, the differences in shear rates for the control and ubiquinol groups were not statistically significant at baseline (747 ± 96 vs. 445 ± 117), HS (775 ± 99 vs. 554 ± 172), and FR (745 ± 103 vs. 462 ± 109).

### Mast cell degranulation

The mast cell degranulation index in the control group was 1.36 ± 0.03 which was significantly higher than the ubiquinol group (1.02 ± 0.01) (*P *<**0.05). A higher mast cell degranulation index (ratio of experimental to control light intensities) indicates a higher degree of mast cell degranulation. These results indicate that mast cell degranulation following FR was reduced by administering ubiquinol.

### Vascular permeability

**Figure 3.**
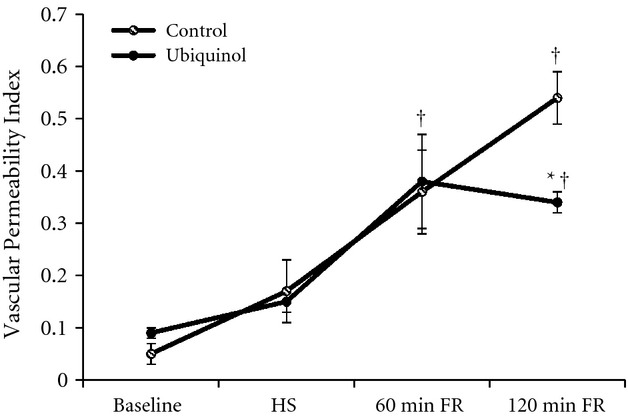
Vascular permeability in the control (*n* = 6) and ubiquinol (*n* = 6) groups following hemorrhagic shock (HS) and fluid resuscitation (FR). The vascular permeability index is the ratio of extra‐ and intravascular FITC‐albumin fluorescence intensity. ^†^Significantly different from baseline (*P *<**0.05). *Significantly different from the control group (*P *<**0.01).

Figure 3 summarizes vascular permeability index, which was calculated as the ratio of extravascular to the intravascular FITC‐albumin fluorescence intensity. The greater the vascular permeability to FITC‐albumin, the higher is the vascular permeability index. At baseline, the vascular permeability indexes in both the control and ubiquinol groups were similar and relatively low (0.05 ± 0.02 vs. 0.09 ± 0.01, respectively, *P *>**0.05). After 1 h of HS, the vascular permeability index increased to 0.17 ± 0.06 in the control group and 0.15 ± 0.02 in the ubiquinol group, respectively, indicating an increase in vascular permeability. The differences in vascular permeability index between the control and ubiquinol groups at HS were not statistically significant (*P > *0.05). Similar results were observed at 1 h after FR in both the control and ubiquinol groups as the vascular permeability indexes continued to increase significantly relative to baseline (0.36 ± 0.08 vs. 0.38 ± 0.09, respectively). Compared to baseline, the vascular permeability index in the control group increased more than 10 times at 2 h after FR (0.54 ± 0.05 vs. 0.05 ± 0.02, *P *<**0.01). Administration of ubiquinol prior to FR significantly reduced the increase in vascular permeability at 2 h after FR to 0.34 ± 0.02, which was significantly less than the control group (0.54 ± 0.05) (*P *<**0.01).

### Reactive oxygen species levels

**Figure 4.**
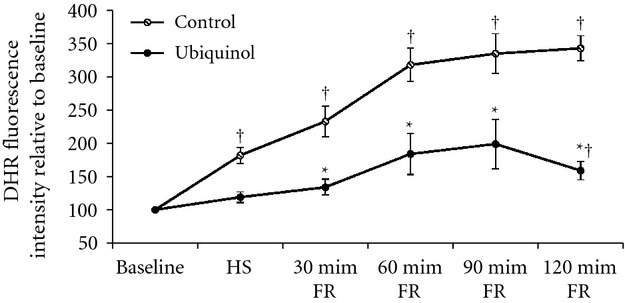
Reactive oxygen species (ROS) levels in the mesenteric venule in the control (*n* = 6) and ubiquinol (*n* = 6) groups following hemorrhagic shock (HS) and fluid resuscitation (FR). Dihydrorhodamine (DHR) fluorescence intensity was used to determine ROS levels. Values of DHR fluorescence intensity at HS and every 30 min post FR were expressed relative to baseline value (100%). ^†^Significantly different from baseline (*P *<**0.01). *Significantly different from the control group (*P *<**0.05).

The cumulative data of ROS levels estimated by DHR fluorescence intensity at baseline, HS, 30‐min, 60‐min, 90‐min, and 120‐min after FR in the control and ubiquinol groups are displayed in Figure 4. The DHR fluorescence intensities at HS and every 30 min after FR were expressed relative to baseline value (100%). The ROS levels in the control group significantly increased compared to baseline following FR (*P < *0.01). The ROS levels in the ubiquinol group at 2 h after FR also significantly increased to 159 ± 35% of baseline (*P *<**0.01); however, it was significantly less than the control group (343 ± 47%) (*P < *0.01).

## Discussion

The major findings from this study are that in rat mesenteric venules administration of ubiquinol significantly: (1) decreased leukocyte adherence; (2) attenuated mast cell degranulation; (3) reduced the increase in vascular permeability; and (4) attenuated the increase in ROS levels following HS and FR.

The determining factors that affect the tendency for leukocytes to adhere to the venular wall are the balance between pro‐adhesive forces and the hydrodynamic dispersal forces. Pro‐adhesive forces include a variety of proinflammatory mediators such as histamine, leukotrienes, and platelet activating factor (PAF) which act on receptors on leukocytes and endothelial cells to promote leukocyte rolling and adhesion (Granger and Senchenkova ^13^). Increased ROS levels from the activated endothelial cells contributes to transcription‐dependent adhesion molecule synthesis and expression (Granger and Kubes ^12^; Panes and Granger ^17^). In contrast, the movement of blood flow acts as an anti‐adhesive force by propelling leukocytes over the endothelial surface. This can be estimated by the venular wall shear rate, which represents the hydrodynamic dispersal force generated at the vessel wall that could interfere with leukocyte adherence. In this study, we did not find significant differences in the shear rates between the control and ubiquinol groups at any time points throughout the experiments. Therefore, the differences in leukocyte adherence between the control and ubiquinol groups may result from the differences in the pro‐adhesive forces. As an antioxidant, ubiquinol decreases ROS levels in the mesenteric microcirculation, thus potentially inhibiting the synthesis and expression of the transcription‐dependent adhesion molecule. Bitencourt et al. found that leukotrienes and platelet activating factor (PAF) are important inflammatory mediators that play critical roles in developing microvascular inflammatory response to ischemia‐reperfusion induced by HS and FR (Bitencourt et al. ^5^). Park et al. found that the release of PAF promoted leukocyte adherence and also stimulated increased generation of ROS especially superoxide from leukocytes. In addition, leukocyte adherence induced by PAF was significantly prevented by superoxide dismutase (Park et al. ^18^). Thus, we suggest administrating ubiquinol before FR may decrease PAF induced leukocyte adherence by decreasing ROS levels. Even so, the sizes of the leukocyte pool in the control and ubiquinol groups were not determined in the study. Therefore, decreased leukocyte adherence in the ubiquinol group may also result from decreased circulating leukocytes, which should be investigated in future studies.

The second significant finding from this study was the ability of ubiquinol to markedly reduce mast cell degranulation following the 2‐h period after FR. Degranulation of mast cells promotes release of various inflammatory mediators (i.e., histamine, serotonin, phospholipases, tryptase etc.) and significantly intensifies the microvascular inflammatory responses (Theoharides et al. ^22^). By preventing mast cell degranulation, reperfusion injury caused by FR following HS could be minimized. Steiner et al. (^20^) observed that cromolyn (a mast cell stabilizer) which blocks mast cell degranulation, resulted in decreased ROS levels, leukocyte adherence, and vascular permeability. In addition, they reported that antioxidants (i.e., lipoic acid) prevented degranulation of mast cells during hypoxia. In our experiments, mesenteric hypoxia was likely present because of decreased hemoglobin concentration and blood flow. Other studies have suggested that an imbalance in the ROS‐nitric oxide is one of the mechanisms involved in mast cell activation and degranulation which contributes to leukocyte endothelial adhesion (Dix et al. ^11^; Swindle and Metcalfe ^21^). Therefore, it is possible that decreased mast cell degranulation found in the present study can be attributed to ubiquinol decreasing ROS levels.

In the study, we observed similar patterns of ischemia‐reperfusion induced increases in vascular permeability to FITC‐albumin in the control group as other investigators (Harris et al. ^14^; Casillan et al. ^7^). Heterogeneous pattern of FITC‐albumin accumulated along the venular wall following HS and FR indicated increased vascular leakage. In general, factors contributing to increased vascular permeability include ultrastructural changes caused by activation of vascular endothelial growth factor (VEGF) and inflammatory mediators such as histamine and serotonin (Bates ^3^). There is evidence showing the involvement of ROS in the underlying mechanisms of alterations in vascular integrity (Laux and Seiffge ^16^). By ubiquinol scavenging excessive ROS, mast cell release of histamine and serotonin is reduced resulting in the attenuation of vascular permeability.

The key finding in our study was that the administration of ubiquinol following HS was effective in significantly decreasing the ROS levels in the mesenteric venular wall. Without ubiquinol, ROS progressively increased throughout the 2‐h period post‐FR thereby producing damage to the microcirculation. During HS/FR, there is systemic hypoxia and alveolar macrophages are activated, which release inflammatory mediators into the circulation causing mast cell degranulation and microvascular inflammation (Chao et al. ^9^). The degranulation of mast cells leads to the release of various inflammatory mediators that further intensifies the inflammatory responses, resulting in increased leukocyte‐endothelial cell adhesive interactions and vascular permeability. The ischemic condition causes the activation of endothelial cells and creates an environment that is prone for ROS generation. During the period of restoring oxygen delivery by returning of blood supply and FR to the ischemic tissue, the activated endothelial cells produce significant amounts of ROS, depleting nitric oxide (Steiner et al. ^19^). The imbalance of ROS levels and nitric oxide leads to escalated inflammatory responses, including increased mast cell degranulation, leukocyte adherence, and increased vascular permeability as observed in the control group. This is termed reperfusion injury. Using ubiquinol, we have demonstrated that it had a significant effect by counteracting the excessive ROS following FR. Furthermore, by controlling the amounts of ROS, ubiquinol was effective in maintaining mast cell stability, decreasing leukocyte‐endothelial cell adhesive interactions, and reducing vascular permeability.

In a previous study, using an identical HS/FR model, we measured mitochondrial superoxide levels in leukocytes, hydrogen peroxide in the diaphragm, and apoptosis in the lung, diaphragm, heart, and kidney. The results indicated animals receiving ubiquinol had significantly reduced levels of ROS and nuclear DNA damage (apoptosis) (Bennetts et al. ^4^). In the present study, ubiquinol attenuated HS and FR induced microvascular inflammation by decreasing ROS levels, leukocyte adherence, mast cell degranulation, and vascular permeability. In conclusion these findings suggest ubiquinol provides protection to the vital organs and microvascular circulation from reperfusion injury occurring after HS and FR.

## Conflict of Interest

None declared.
